# Buddi-Chiari syndrome associated with hypereosinophilic syndrome: A case report

**DOI:** 10.1097/MD.0000000000034291

**Published:** 2023-08-11

**Authors:** Zhaoxia Li, Nan Li, Zhuhui Ji, Jiahe Shi, Guijie Xin

**Affiliations:** a Department of Hepatology, The First Hospital of Jilin University, Jilin Province, China; b Center for Pathogen Biology and Infectious Disease, The First Hospital of Jilin University, Changchun, Jilin Province, China.

**Keywords:** Buddi-Chiari syndrome, hypereosinophilic syndrome, JAK2V617F

## Abstract

Budd-Chiari Syndrome (BCS) is a relatively rare clinical disorder with a wide range of symptoms, caused by the obstruction of the hepatic venous outflow. The etiology and pathogenesis of BCS vary in different countries and regions. In Western countries, hepatic venous obstruction is the most common type, and its main cause is closely related to the hypercoagulable state of the body. Inferior vena cava obstruction is common in Asia, and its etiology progresses slowly due to the lack of epidemiological data. [3] Here, we report a rare case of BCS associated with the hypereosinophilic syndrome and discuss the possible causal relationship between the two.

**Patient concerns:**

The patient was a 33-year-old female with intermittent epistaxis, gum bleeding, and excessive menstrual flow for the past 6 months. The routine blood tests showed elevated levels of eosinophils, and the liver function test showed mildly elevated levels of γ-glutamyl transpeptidase and alkaline phosphatase, and abdominal ultrasound showed hepatosplenomegaly and suspicion of intrahepatic arteriovenous or arteriovenous-portal fistula.

**Diagnoses:**

Finally, through the improvement of bone marrow aspiration, digital subtraction angiography and gene detection, the diagnosis of BCS combined with hypereosinophilic syndrome was confirmed, and JAK2V617F mutation was highly associated with it.

**Interventions:**

The patient received endovascular stent implantation and regular oral rivaroxaban anticoagulation therapy after operation.

**Outcomes:**

Seven months later, enhanced computed tomography (CT) of the hepatobiliary showed that the hepatic bruise-like changes were significantly reduced compared with before, and the right hepatic vein and the right perihepatic vein stent were left in place with a good filling of contrast in the stent.

**Lessons:**

The patient, in this case, was finally diagnosed with BCS combined with hypereosinophilic syndrome, and to our knowledge, such case reports are rare. Our case report suggest an association between BCS and hypereosinophilic syndrome, but relevant studies are minimal, we hope to conduct larger and higher quality studies on these patients in the future, to provide new directions and basis for the etiology and pathogenesis of these diseases, as well as provide new targets and ideas for clinical treatment.

## 1. Introduction

Buddi-Chiari Syndrome (BCS) is a clinical syndrome of obstruction of the hepatic outflow tract at any level from the small hepatic vein to the connection of the inferior vena cava with the right atrium, followed by the portal and/or inferior vena cava hypertension, which can be classified as hepatic venous obstruction, inferior vena cava obstruction, or mixed type according to the anatomical location.^[1,2]^ In Western countries, hepatic venous obstruction is the most common type, and its main cause is closely related to the hypercoagulable state of the body. Inferior vena cava obstruction is common in Asia, and its etiology progresses slowly due to the lack of epidemiological data.^[3]^ Here, we report a case of BCS associated with hypereosinophilic syndrome and discuss the clinical significance of etiological studies of BCS and hypereosinophilic syndrome and their relevance to treatment strategies.

## 2. Case report

**Figure 1.**
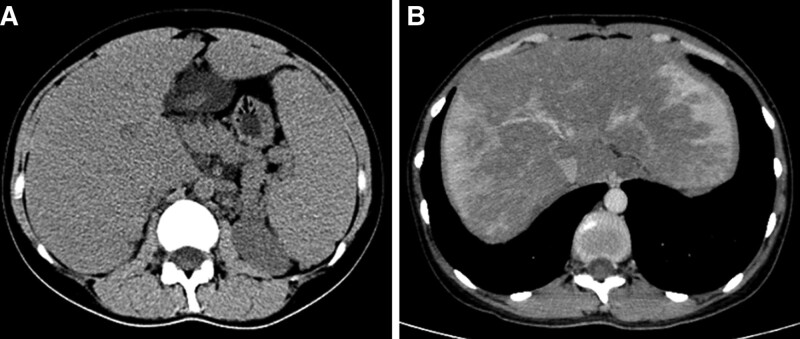
Hepatobiliary CT scan + enhancement. (A) Full liver morphology, splenomegaly. (B) Uneven density of hepatic veins. CT = computed tomography.

The patient was a 33-year-old female with intermittent epistaxis, gum bleeding, and excessive menstrual flow for the past 6 months. The routine blood tests showed elevated levels of eosinophils, and the liver function test showed mildly elevated levels of γ-glutamyl transpeptidase and alkaline phosphatase, and abdominal ultrasound showed hepatosplenomegaly and suspicion of intrahepatic arteriovenous or arteriovenous-portal fistula. The gynecologic ultrasound did not show any significant abnormalities, and the patient was admitted to our hospital for further treatment. The patient enlarged liver and spleen could be palpated on physical examination. There was no abdominal pain, but there was abdominal distension and it was progressively worsening. The patient had previous allergic purpura for 9 years, splenomegaly was found for 7 years, there was a history of bronchial asthma for 3 years, and no recent asthma attacks. A review of the patient past cases revealed that the patient had developed eosinophilia and splenomegaly as early as 2013, and no hepatomegaly was seen. Routine blood count on admission: eosinophil percentage 0.147, absolute eosinophil value 1.05 × 10^9^/L; Coagulation routine: prothrombin time 16.5 seconds, international normalized ratio 1.38, prothrombin time activity 57%; Liver function: γ‐glutamyl transpeptidase 158.0U/L, alkaline phosphatase 101.5U/L; hypersensitive C-reactive protein, procalcitonin, ions, urine routine, stool examination, infection markers, iron metabolism, ceruloplasmin, blood lipids, fasting blood glucose, tumor markers, IgG antibodies in autoimmune liver disease, renal function and femoral X-ray were all normal. Hepatobiliary computed tomography (CT) scan + enhancement (Fig. 1A and B) returned: full liver morphology, splenomegaly, hepatic bruise-like changes, and hepatic vein density heterogeneity. Portal ultrasound indicates suspicion of intrahepatic arteriovenous or arteriovenous-portal fistula; inferior vena cava ultrasound suggested increased flow velocity in the post-hepatic segment of the inferior vena cava. No clear signs of thrombosis were seen, which seems to be more suggestive of the diagnosis of hepatic arteriovenous fistula.

An intrahepatic arteriovenous fistula is defined as the presence of an abnormal shunt channel between the hepatic artery and the portal vein or hepatic vein. The disease is mostly due to congenital vascular anomalies, but can also occur secondary to liver trauma and medical manipulation, etc. The portal blood flow increases significantly, which can lead to the manifestation of portal hypertension such as the opening of collateral circulation, ascites, and hydrothorax.^[4]^ However, no signs of arteriovenous fistula were seen on enhanced CT, but the hepatic veins appeared to have a filling defect, and further digital subtraction angiography was performed to clarify the presence of hepatic vascular disease. Before this examination, we noted that the patient eosinophils were still high, and the cytology of the bone marrow smear suggested an increase in the proportion of eosinophils in the bone marrow and peripheral blood. The bone marrow biopsy suggested granulocytosis, erythropoiesis, and macrophage hyperplasia with eosinophilia.

**Figure 2.**
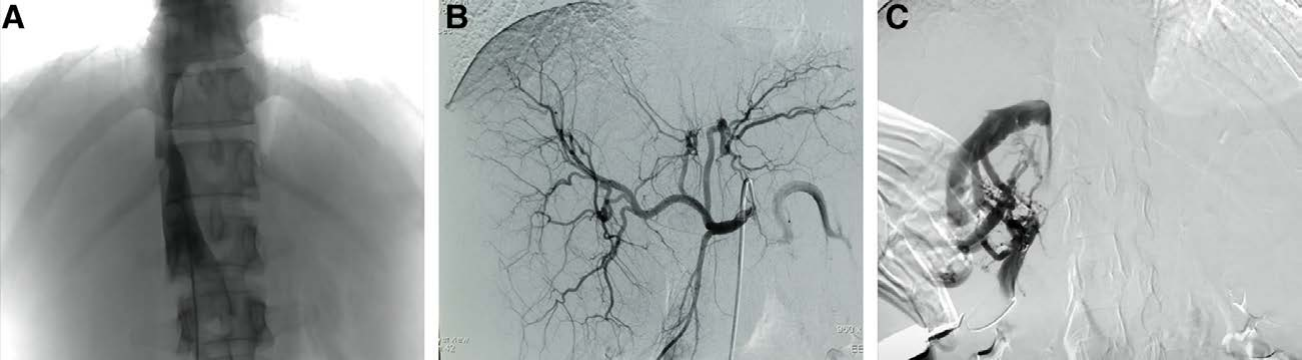
DSA examination. (A) Slight thinning of the inferior vena cava at the hepatic level. (B) No intrahepatic arteriovenous or arteriovenous-portal fistula. (C) Occlusion of the hepatic vein at its confluence with the inferior vena cava. DSA = digital subtraction angiography.

**Figure 3.**
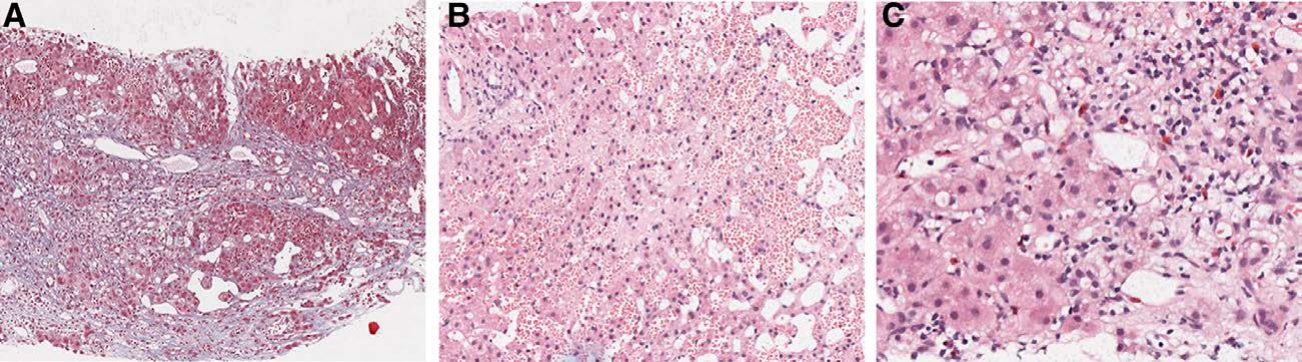
Liver biopsy. (A) The structure of hepatic acinus was disordered, the portal areas showed obvious dilatation and fibrosis, bridging fibrosis, nodular hyperplasia of residual hepatocytes. (B) The hepatic sinusoids were markedly dilated and congested; the area around the central vein was most pronounced, the hepatocytes were atrophic to varying degrees. (C) More lymphocytes and scattered eosinophils infiltration in the interstitium.

We first performed a venogram (Fig. 2A),which showed a slight thinning of the inferior vena cava at the hepatic level and no opening in the hepatic vein was found. Further hepatic arteriography (Fig. 2B) was performed, which showed no obvious abnormalities in the hepatic artery or branches, and no intrahepatic arteriovenous or arteriovenous-portal fistula was observed. We continued to look for the hepatic vein at the third hepatic portal (Fig. 2C) and saw that the hepatic vein was occluded at the confluence of the hepatic vein into the inferior vena cava, and the inferior vena cava did not show up, which was consistent with the imaging presentation of BCS-hepatic vein obstruction type. And we completed the liver biopsy intraoperatively. It is worth mentioning that because of the patient hypersensitivity, the operator applied dexamethasone intraoperatively. Postoperative blood monitoring without hormone application observed a rapid decline in eosinophils followed by a gradual rebound. During this period, the patient liver biopsy results (Fig. 3A–C) returned: extensive hepatocyte loss and fibrosis mainly around the central vein, marked dilatation and bruising of the surrounding hepatic sinusoids, and moderate lymphocytic and eosinophilic infiltration in the interstitium; consistent with BCS. The patient was finally diagnosed with BCS and hypereosinophilic syndrome. She was given symptomatic supportive treatment and endovascular stent implantation, and regular oral rivaroxaban anticoagulation therapy for 7 months after surgery. The liver function, blood routine and coagulation routine were normal, and no hemostatic and thrombotic disease genetic variants highly correlated with his clinical phenotype were found. Enhanced CT of the hepatobiliary showed that the hepatic bruise-like changes were significantly reduced compared with before, and the right hepatic vein and the right perihepatic vein stent were left in place with a good filling of contrast in the stent.

## 3. Discussion

As a rare disease, there is a lack of epidemiological information on a global scale for BCS.A meta-analysis^[5]^ combining a total of 6 high-quality studies in Asian and European populations showed that the prevalence of BCS ranged from 0.168/1 million to 4.09/1 million, and that the overall prevalence in Asia was lower than in Europe. The etiology and pathogenesis of BCS also vary in different countries and regions. The hepatic venous obstruction type is predominant in Western countries, with approximately 80% of patients having at least one risk factor for thrombosis. A series of hypercoagulable states of the blood cause thrombosis, followed by narrowing or even occlusion of the lumen, of which myeloproliferative neoplasms are the most common cause.^[6,7]^ A meta-analysis^[8]^ included 19 European studies with a total of 1062 patients, with a prevalence of 40.9% for myeloproliferative neoplasms and 41.1% for the associated JAK2V617F positive mutation, suggesting routine screening for this gene in patients with BCS. In contrast, there are significant geographical differences in the epidemiology of BCS in the Asia-Pacific region, and reliable population-based epidemiological information is lacking. In addition to the above mentioned factors, oral contraceptives and pregnancy can also induce the development of BCS. The current gold standard for the diagnosis of BCS is angiography, which can show the degree of obstruction and the pressure in each vessel, but it is not routinely performed as an invasive test and is costly. Color Doppler ultrasonography is low cost, simple to perform, shows thrombosis, distal luminal stenosis, and secondary proximal luminal dilatation, and also evaluates changes in blood flow, and is often the preferred screening method. Enhanced CT is another commonly used test that shows filling defects in the inferior vena cava and hepatic veins. Magnetic resonance imaging is mainly used to show the open state of the hepatic veins/inferior vena cava and changes in the liver parenchyma. Liver biopsy is no longer a routine test, and only the diagnosis of small vessel BCS depends on liver biopsy.^[3]^

Hypereosinophilic syndrome presents with unexplained persistent elevation of blood and/or bone marrow eosinophils, and according to the 2012 multidisciplinary consensus,^[9]^ the diagnosis of hypereosinophilic syndrome requires: an absolute eosinophil count of >1.5 × 10^9^/L in peripheral blood on 2 tests (>1 month apart) and/or a bone marrow eosinophil percentage ≥20% and/or Pathologically confirmed extensive eosinophil infiltration and/or significant eosinophil granule protein deposition. They are mainly classified as reactive, neoplastic/myeloproliferative and idiopathic eosinophilia^[10]^: eosinophilia is considered reactive if it is cytokine-driven, ranging from inflammatory diseases to parasitic infections; neoplastic/myeloproliferative eosinophilia is considered if clonal eosinophilia indicators such as PDGFRA and PCM-JAK2 rearrangement are detected; the diagnosis of idiopathic eosinophilia can only be made after the above 2 exclusions. Eosinophils are multifunctional leukocytes that can protect the body from parasites by degranulation and release of interleukin-5 (IL-5) cytokines, as well as induce tissue damage and dysfunction by secreting a range of cytotoxic granules and neurotoxins.^[11,12]^ Valente et al^[13]^ suggested that eosinophilia is due to the secretion of cytokines such as IL-5,granulocyte-macrophage colony stimulating facto, and IL-3 by specific monoclonal T cells, mast cells, and stromal cells, which also activate tyrosine kinases and further reactivate the Ras-MAPK and JAK signaling pathways, which together mediate the differentiation, migration, and activation of eosinophils. The tissue damage associated with eosinophilia is also due to the direct infiltration of eosinophils and their indirect effects such as the release of proteases, inflammatory factors, and other cytokines. Only a few reports^[14,15]^ have suggested a causal relationship between hypereosinophilic syndrome and BCS, presumably by a mechanism of eosinophil infiltration into the inferior vena cava and/or hepatic vein wall, releasing cationic proteins, peroxidases, and eosinophil-derived neurotoxins, etc, causing endothelial damage and hypercoagulability, which in turn leads to thrombosis.

We analyzed the etiology of the patient BCS. First, we learned that the patient had taken oral short-acting contraceptives several times in the past 10 years and had 1 pregnancy, which was aborted because the fetus stopped developing. Antiphospholipid syndrome is a series of syndromes with clinical manifestations of arteriovenous thrombosis, pathological pregnancy, or thrombocytopenia mediated by antiphospholipid antibodies, and it is also one of the causes of BCS. Here, we considered the patient repeated use of short-acting contraceptives as one of the risk factors for BCS. In addition, we performed a JAK2V617F gene test for this patient, which showed a positive mutation in 10.24% of the quantitative tests. JAK2V617F mutation leads to substitution of phenylalanine by valine at position 617, which in turn causes cytokine hypersensitivity.^[16]^ This mutation is very common in patients with polycythemia vera, primary thrombocythemia, and idiopathic myelofibrosis, but is rare in hypereosinophilic syndrome.^[17]^ Only a few case reports have described hypereosinophilic syndrome associated with the JAK2V617F mutation.^[17–21]^ It has been shown that GM-CSF and IL-5 transmit anti-apoptotic signals in eosinophils,^[22]^ Itsuo Iwamoto et al^[23]^ further found that activation of the JAK2-STAT5 pathway plays a key role in the anti-apoptotic signaling of GM-CSF in human eosinophils, and that the JAK2 blocker AG-490 inhibits JAK2 phosphorylation and eosinophil survival via GM-CSF. In this regard, we conjecture that the JAK2-V617F mutation leads to activation of the JAK2-STAT5 pathway, which in turn increases eosinophilia and causes vascular damage as another risk factor for the development of BCS in this patient. At the same time, this patient suggests to us whether testing for JAK2V617F mutations in Asian populations should be increased, especially in patients without evidence of myeloproliferative neoplasms.

In terms of treatment, the 2021 APASL guidelines^[3]^ suggest that: first, all patients should receive anticoagulation therapy, and low molecular weight heparin, warfarin and direct oral anticoagulants have good efficacy. In addition, we can choose angioplasty such as thrombolysis, balloon dilation, and stent placement to correct hepatic outflow tract obstruction according to individualized principles. TIPS is the treatment of choice when the above treatments are not effective. Liver transplantation is the treatment of last resort, and anticoagulation is recommended after liver transplantation. Treatment of hypereosinophilic syndrome^[24]^: In critically ill patients, especially those with leukocyte stasis, or other serious complications, single high-dose glucocorticoid shock therapy should be given as soon as possible if parasitic infection is excluded, and if this cannot be ruled out, ivermectin should be used in combination to prevent fatal superinfection syndrome. For non-serious patients: the tyrosine kinase inhibitor imatinib is preferred if the disease is neoplastic/myeloproliferative eosinophilia. If the disease is reactive and idiopathic eosinophilia, no treatment is needed if asymptomatic, glucocorticoid therapy is preferred if symptomatic, and second-line therapy, such as hydroxyurea, imatinib, interferon-alpha, or mepolizumab, can be considered if the outcome is poor.

In conclusion, for patients with unexplained hepatosplenomegaly, we need to start with a medical history and screen for multiple aspects. The patient, in this case, was finally diagnosed with BCS combined with hypereosinophilic syndrome, and to our knowledge, such case reports are rare. We hope to conduct larger and higher quality studies on these patients in the future, to provide new directions and basis for the etiology and pathogenesis of these diseases, as well as providing new targets and ideas for clinical treatment.

## Author contributions

**Funding acquisition:** Guijie Xin.

**Investigation:** Zhuhui Ji, Jiahe Shi.

**Resources:** Guijie Xin.

**Supervision:** Guijie Xin.

**Validation:** Guijie Xin.

**Writing – original draft:** Zhaoxia Li, Nan Li.

**Writing – review & editing:** Zhaoxia Li, Nan Li.
